# Oncological outcomes of visibly complete transurethral resection prior to neoadjuvant chemotherapy for bladder cancer

**DOI:** 10.1590/S1677-5538.IBJU.2023.0123

**Published:** 2023-05-20

**Authors:** Bryce Baird, Ahmet Bilgili, Augustus Anderson, Gianpiero Carames, Ram A. Pathak, Colleen T. Ball, Raymond Pak, Andrew Zganjar, Paul R. Young, Timothy D. Lyon

**Affiliations:** 1 Mayo Clinic Department of Urology Jacksonville FL USA Department of Urology Mayo Clinic, Jacksonville, FL, USA; 2 Tulane University School of Medicine New Orleans LA USA Tulane University School of Medicine, New Orleans, LA, USA; 3 University of Alabama Department of Pathology Birmingham AL USA Department of Pathology, University of Alabama at Birmingham, Birmingham, AL, USA; 4 Mayo Clinic Department of Quantitative Health Sciences Jacksonville FL USA Department of Quantitative Health Sciences, Mayo Clinic, Jacksonville, FL, USA

**Keywords:** Cystectomy, Neoadjuvant Therapy, Transurethral Resection of Bladder

## Abstract

**Purpose:**

To evaluate the potential oncologic benefit of a visibly complete transurethral resection of a bladder tumor (TURBT) prior to neoadjuvant chemotherapy (NAC) and radical cystectomy (RC).

**Materials and Methods:**

We identified patients who received NAC and RC between 2011-2021. Records were reviewed to assess TURBT completeness. The primary outcome was pathologic downstaging (<ypT2N0), with complete pathologic response (ypT0N0) and survival as secondary endpoints. Logistic regression and Cox proportional hazards models were utilized.

**Results:**

We identified 153 patients, including 116 (76%) with a complete TURBT. Sixty-four (42%) achieved <ypT2N0 and 43 (28%) achieved ypT0N0. When comparing those with and without a complete TURBT, there was no significant difference in the proportion with <ypT2N0 (43% vs 38%, P=0.57) or ypT0N0 (28% vs 27%, P=0.87). After median follow-up of 3.6 years (IQR 1.5-5.1), 86 patients died, 37 died from bladder cancer, and 61 had recurrence. We did not observe a statistically significant association of complete TURBT with cancer-specific or recurrence-free survival (p≥0.20), although the hazard of death from any cause was significantly higher among those with incomplete TURBT even after adjusting for ECOG and pathologic T stage, HR 1.77 (95% CI 1.04-3.00, P=.034).

**Conclusions:**

A visibly complete TURBT was not associated with pathologic downstaging, cancer-specific or recurrence-free survival following NAC and RC. These data do not support the need for repeat TURBT to achieve a visibly complete resection if NAC and RC are planned.

## INTRODUCTION

The use of cisplatin-based neoadjuvant chemotherapy (NAC) has led to improved survival in patients with muscle-invasive bladder cancer (MIBC), with a 5-6.5% benefit in 5-year survival compared to patients who receive radical cystectomy (RC) alone (1–3). Accordingly, use of NAC is endorsed by clinical practice guidelines for all cisplatin-eligible patients with MIBC prior to undergoing RC (4, 5). However, although transurethral resection of a bladder tumor (TURBT) plays an essential role in the management of bladder cancer patients, the importance of achieving a visibly complete TURBT for patients who go on to receive NAC and RC remains unclear. Frequently, tumor characteristics may preclude a visibly complete resection with a single TURBT, or a patient referred to a tertiary center for RC may arrive after having undergone an incomplete TURBT elsewhere. For such patients, it is not known whether the benefit of a repeat TURBT to eradicate all visible tumor outweighs the risks associated with delaying the start of chemotherapy.

The existing literature regarding the potential oncological benefits of a complete TURBT before NAC and RC are conflicting, and uncertainty remains regarding the importance of ensuring complete TURBT prior to initiation of systemic therapy. McKiernan and colleagues evaluated their institutional series and observed that a complete TURBT was associated with significantly higher rates of pathologic downstaging, and cancer-specific survival compared to those without (6). Conversely, Lotan and colleagues found no difference in pathologic or survival outcomes when examining patients with and without complete TURBT before NAC and RC in their practice (7). Thus, the oncologic importance of a visibly complete TURBT before NAC and RC remains unclear, and further data are needed to help clarify this clinical question.

Against the backdrop of this conflicting literature, we hypothesized that a visibly complete TURBT would be associated with pathologic downstaging and improved cancer-specific survival following NAC and RC. Therefore, the objective of this study was to evaluate the association of a visibly complete TURBT with pathologic downstaging and survival among patients treated with NAC and RC at our institution.

## MATERIAL AND METHODS

### Study Cohort

After institutional review board approval (IRB 20-004898), we conducted a retrospective cohort study of patients who underwent RC at Mayo Clinic Florida between May 2011 and May 2021 by one of two surgeons (P.R.Y. and T.D.L.). Inclusion criteria consisted of patients treated with at least one cycle of cisplatin-based NAC followed by RC for nonmetastatic, muscle-invasive bladder cancer. Excluded patients were those treated with upfront RC or who received non-cisplatin containing NAC regimens. Additionally, we excluded patients with a clinical complete response to NAC who refused immediate cystectomy and were observed, but who ultimately underwent RC for a metachronous bladder recurrence (n=5).

### Exposure and Outcomes

Surgical records were individually reviewed by a single investigator (A.A.) to ascertain completeness of TURBT. A procedure was considered visibly complete if the operative report suggested all visible intraluminal tumor was resected down to the level of the bladder wall. Evidence of residual unresected papillary tumor at completion of procedure was considered an incomplete resection. Criteria to perform a second TURBT were not standardize, and this was pursued at each surgeon's discretion for situations such as high grade T1 on first resection or concern for incomplete initial resection. If a second TURBT was performed, patients were classified as having undergone complete TURBT if the second TURBT was visibly complete. If there was no comment on visual complete resection in the operative report, these patients were classified as having undergone an incomplete resection (n=6). Clinical staging was determined via review of operative reports, evaluation of TURBT specimens, physical examination as documented in medical records, and cross-sectional imaging studies.

The primary outcome was pathologic downstaging at RC, defined as <ypT2N0 disease on pathologic specimen. All specimens were reviewed by a fellowship-trained genitourinary pathologist and reported according to the American Joint Committee on Cancer TNM staging, 8th edition (8). Secondary outcomes included complete pathologic response (ypT0N0), recurrence-free survival (RFS), cancer-specific survival (CSS), and overall survival (OS). Survival was estimated starting from date of RC. Death was attributed to cancer when preceded by a documented radiographic or biopsy-proven cancer recurrence.

### Statistical Analysis

Continuous variables were summarized with median and range, and categorical variables were summarized with frequency and percentages. We explored differences in characteristics between those with visibly complete TURBT and those without visibly complete TURBT using Wilcoxon rank sum test for numeric variables and Fisher's exact test for categorical variables.

In evaluation of the primary outcome, associations of TURBT completeness with pathologic outcomes at RC were examined with single variable and multivariable logistic regression, where odds ratios (OR) and 95% confidence intervals (CIs) were estimated. Multivariable logistic regression models were developed by first fitting the model with TURBT completeness and all demographics, TURBT information, and chemotherapy information from Table-1 as covariates in the model, removing the variable with the highest p-value using a backward selection approach while keeping TURBT completion in the model, and refitting the model until there was no longer a reduction in the Akaike Information Criterion. Akaike Information Criterion is an estimator of prediction error and provides an assessment for how well a statistical model fits the data it was generated from, and can be used to compare different model iterations to identify the “best” model for the data set that explains the greatest amount of data variation with fewest number of candidate predictors (9).

**Table 1:** Patient Characteristics.

Variable		N	All patients (N=153)	Visibly Incomplete TURBT (N=37)^a^	Visibly Complete TURBT (N=116)^a^	P^b^
**Demographics and initial TURBT information**						
	Age, years	150	69 (35, 83)	71 (48, 83)^3^	69 (35, 83)	0.24
	Male gender	153	125 (82%)	28 (76%)	97 (84%)	0.33
	BMI, kg/m^2^	153	26.3 (16.3, 44.5)	25.9 (16.8, 42.2)	26.5 (16.3, 44.5)	0.10
	ECOG status ≥ 1	152	55 (36%)	18 (50%) ^1^	37 (32%)	0.07
	Preoperative hydronephrosis	149	100 (67%)	19 (56%) ^3^	81 (70%) ^1^	0.15
**Clinical stage**		**150**				**0.17**
	Ta/Tis/T1		33 (22%)	4 (12%) ^3^	29 (25%)	
	T2		74 (49%)	17 (50%) ^3^	57 (49%)	
	T3 or T4		43 (29%)	13 (38%) ^3^	30 (26%)	
Pure Urothelial Histology		153	127 (83%)	30 (81%)	97 (84%)	0.80
Second TURBT		149	58 (39%)	9 (26%) ^3^	49 (43%) ^1^	0.11
**Chemotherapy information**						
Cycles of chemotherapy		148	3 (1, 6)	3 (1, 4) ^3^	3 (1, 6) ^2^	0.49
4+ cycles		148	67 (45%)	13 (38%) ^3^	54 (47%) ^2^	0.43
**Type of NAC**		**149**				**0.27**
	Gem/Cis		115 (77%)	30 (86%) ^2^	85 (75%) ^2^	
	MVAC		26 (17%)	3 (9%) ^2^	23 (20%) ^2^	
	Other *		8 (5%)	2 (6%) ^2^	6 (5%) ^2^	
**Cystectomy information**						
	Age, years	153	70 (36, 84)	72 (48, 84)	70 (36, 83)	0.25
	Days from initial TURBT to cystectomy	150	175 (52, 534)	164 (52, 395) ^3^	178 (68, 534)	0.52
**Diversion type**		**152**				**0.10**
	Ileal conduit		104 (68%)	31 (84%)	73 (63%) ^1^	
	Orthotopic neobladder		36 (24%)	4 (11%)	32 (28%) ^1^	
	Right colon pouch		11 (7%)	2 (5%)	9 (8%) ^1^	
	Cutaneous ureterostomy		1 (1%)	0 (0%)	1 (1%) ^1^	
**Approach of surgery**		**152**				**0.05**
	Open		49 (32%)	17 (46%)	32 (28%) ^1^	
	Robotic		103 (68%)	20 (54%)	83 (72%) ^1^	
**Pathologic T stage**		**153**				**0.64**
	T0		46 (30%)	10 (27%)	36 (31%)	
	Ta/Tis/T1		23 (15%)	5 (14%)	18 (16%)	
	T2		29 (19%)	5 (14%)	24 (21%)	
	T3		33 (22%)	11 (30%)	22 (19%)	
	T4		22 (14%)	6 (16%)	16 (14%)	
Pathologic N+		153	38 (25%)	7 (19%)	31 (27%)	0.39
Positive soft tissue margin		153	16 (10%)	3 (8%)	13 (11%)	0.76

The sample median (minimum, maximum) is shown for numeric variables. Number (percentage of patients) is shown for categorical variables.

* Other: includes cisplatin/etoposide and cisplatin/paclitaxel;

a Superscript numbers represent the number of patients with unavailable information.;

b P values result from the Wilcoxon rank sum test for continuous data and Fisher's exact test for categorical data.

The reverse Kaplan-Meier method was used to estimate the median (interquartile range; IQR) length of follow-up after cystectomy where patients who died were censored at their death date. Survival outcomes were graphically depicted using the Kaplan-Meier method and compared using the log-rank test. Associations of TURBT completeness with RFS, CSS and OS after cystectomy were examined with single variable and multivariable Cox proportional hazards regression, where hazard ratios (HR) and corresponding 95% Cis were estimated. Multivariable Cox proportional hazards regression models were developed in a similar approach as the logistic regression models using backward candidate variable selection; however, cystectomy information was also considered for inclusion in models of survival outcomes after RC. Pathologic T stage was retained in all survival models. In all multivariable models, missing data were imputed with either the sample median or the most frequent category. P-values less than 0.05 were considered statistically significant without adjustment for multiple testing. All statistical tests and confidence intervals were two-sided. Statistical analyses were performed using R (version 4.1.2; R Foundation for Statistical Computing, Vienna, Austria).

## RESULTS

We identified 153 patients treated with NAC and RC between 2011-2021, including 116 (76%) with a visibly complete TURBT prior to NAC. Of the patients under study, 42% (64/153) experienced pathologic downstaging and 28% (43/153) exhibited a complete pathologic response at RC. Median follow-up after RC was 3.6 years (IQR 1.5-5.1 years), during which time 86 patients had recurrence or died (62 died and 61 experienced disease recurrence).

Patient characteristics and clinical information are shown in Table-1. A second TURBT was performed in 58 patients; 11 of whom had visibly incomplete resections on both TURBTs. Thirteen patients had variant histology, including micropapillary (n=7), small cell (n=3), sarcomatoid (n=1), adenocarcinoma (n=1), and squamous cell carcinoma (n=1).

Pathologic response outcomes at RC are shown in Table-2. There was no significant difference in the likelihood of pathologic downstaging at RC (<ypT2N0) for those with visibly incomplete compared to complete TURBT (38% (14/37) versus 43% (50/116), P=0.57). The absolute difference in the proportion of patients who had pathologic downstaging at RC was 5% higher among those with complete TURBT compared to incomplete TURBT, but this was not statistically significant after adjustment for potentially confounding variables (adjusted OR 0.97, 95% CI 0.42-2.26, P=0.95). Likewise, we did not find evidence of an association of incomplete versus complete TURBT with complete pathologic response at RC (ypT0N0), (27% (10/37) versus 28% (33/116), P=0.87), even after multivariable adjustment (adjusted OR 1.24, 95% CI 0.49 – 3.01, P=0.64). Supplemental Table-1 shows the coefficients from the multivariable logistic regression models.

**Table 2:** Association of TURBT completeness with pathologic and survival outcomes after cystectomy.

Outcome		Incomplete TURBT (N=37), n (%)	Complete TURBT (N=116), n (%)	Unadjusted OR or HR (95% CI), Incomplete vs. Complete TURBT	P	Adjusted OR or HR (95% CI), Incomplete vs. Complete TURBT	P
**Pathologic response at cystectomy**							
	Downstaging (<pT2)	14 (38%)	50 (43%)	0.80 (0.37-1.70)	0.57	0.97 (0.42-2.26) a	0.95
	Complete response (pT0)	10 (27%)	33 (28%)	0.93 (0.39-2.09)	0.87	1.24 (0.49-3.01) b	0.64
**Survival outcomes after cystectomy**						
	Recurrence	12 (32%)	49 (42%)	0.92 (0.49-1.74)	0.81	0.90 0.47-1.71) c	0.74
	Cancer-specific death	11 (30%)	26 (22%)	1.59 (0.78-3.21)	0.20	1.47 (0.70-3.10) d	0.31
	Death, any cause	22 (59%)	40 (34%)	1.96 (1.16-3.31)	0.012	1.77 (1.04-3.00) e	0.034

Odds ratios (OR), 95% confidence intervals (CI) and p-values were estimated from logistic regression models for evaluating the associations of incomplete TURBT vs. complete TURBT with pathologic response at cystectomy.

Hazard ratios (HR), 95% confidence intervals (CI) and p-values were estimated from Cox proportional hazards regression models for evaluating the associations of incomplete TURBT vs. complete TURBT with survival outcomes after cystectomy.

a Adjusted for age at cystectomy, ECOG 1+, and preoperative hydronephrosis.

b Adjusted for sex and preoperative hydronephrosis.

c Adjusted for age at cystectomy, body mass index, days from initial TURBT to cystectomy (log scale), and pathologic T stage.

d Adjusted for age at cystectomy, sex, body mass index, ECOG 1+, preoperative hydronephrosis, second TURBT, days from initial TURBT to cystectomy (log scale), pathologic T stage, and soft tissue margin.

e Adjusted for ECOG 1+ and pathologic T stage.

**Figure 1:**
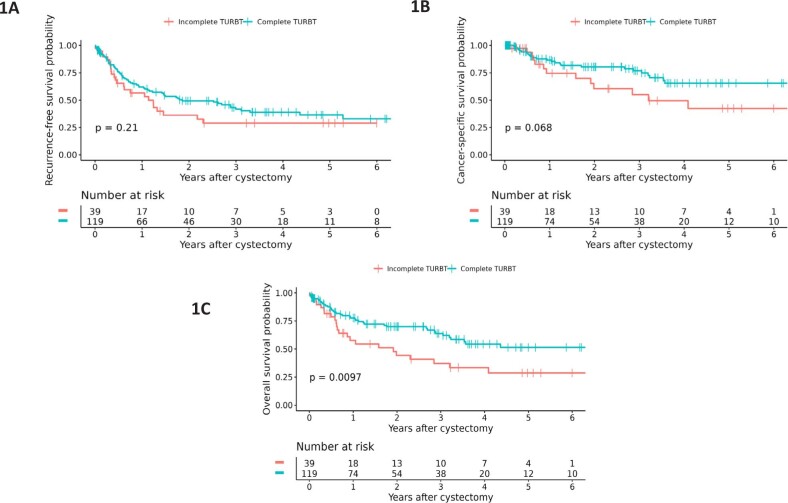
Kaplan-Meier estimates of (A) recurrence-free, (B) cancer-specific, and (C) overall survival following neoadjuvant chemotherapy and radical cystectomy, stratified by completeness of initial TURBT.

Kaplan-Meier estimates of RFS, CSS and OS according to TURBT completeness are shown in Figure-1 and results of Cox proportional hazards models in Table-2. Following multivariable adjustment there was no statistically significant association between TURBT completeness and RFS (adjusted HR 0.90, 95% CI 0.47-1.71, P=0.74) or CSS (adjusted HR 1.47, 95% CI 0.70-3.10, P=0.31). However, we did observe that the hazard of death from any cause was higher among those who had visibly incomplete TURBT (adjusted HR 1.77, 95% CI 1.04-3.00, P=0.034). Supplemental Table-2 shows the coefficients from the multivariable models for the survival related outcomes. Kaplan-Meier estimates of RFS, CSS and OS stratified by pathologic stage are shown in the Supplemental Figure, confirming more favorable survival outcome among patients with lower pathologic stage.

## DISCUSSION

Herein, we examined our institutional series of patients treated with NAC and RC over a 10-year period and did not find evidence of an association of visibly complete TURBT with pathologic response at RC. This lack of a significant association persisted after adjusting for multiple relevant confounders. Similarly, we did not observe differences in RFS and CSS between those with and without a complete TURBT. While we did find longer OS among those with a complete TURBT, this is mostly likely attributable to residual unmeasured medical confounders impacting the decision to pursue a complete TURBT given that pathologic outcomes, RFS and CSS were similar. We continue to advocate for an attempt at visibly complete initial TURBT, in accordance with clinical practice guidelines, for all patients in whom the presence or absence of muscle invasion is not yet known (10). However, the data reported here contribute to a growing body of evidence that does not support the need for a repeat TURBT solely to achieve a visibly complete resection in a patient referred with known MIBC in whom NAC and RC are already planned.

TURBT plays an important prognostic role for MIBC patients who undergo RC and contributes to pathological downstaging. Indeed, TURBT alone can render approximately 15% of patients pT0 on final pathology, which is further increased to around 40% in combination with cisplatin-based NAC (11). Achieving pT0 status remains associated with an excellent prognosis after RC, although those achieving ypT0 after NAC has been shown to be associated with a worse prognosis as compared to those with pT0 after TURBT alone (12–14). Brant and colleagues compared the adjusted relative risk of pathologic response at RC between patients treated with and without NAC, and estimated that 38% of pathological response seen at RC among patients receiving NAC can be attributed to the TURBT alone (15). This finding suggests a potential theoretical benefit to complete TURBT, as patients with residual cancer at RC following NAC are known to have worse prognosis compared to those with residual disease after TURBT alone (16).

Nevertheless, clinical series have not reliably supported the need for a complete TURBT prior to NAC and RC and have reported conflicting findings, which complicates decision-making for clinicians. Bree and colleagues recently evaluated their contemporary institutional series of 548 MIBC patients and used propensity scores to match patients treated with and without repeat TURBT prior to RC (17). They observed that absence of disease on repeat TURBT was associated with an improved prognosis, but that there was no difference in survival outcomes irrespective of the use of NAC. Researchers at the University of Washington found that patients who underwent maximal TUR for MIBC patients was not significantly associated with pT0 after multivariable adjustment, including use of NAC (18). In a study from the University of Texas Southwestern, Ghandour and colleagues found that grossly complete TURBT was not significantly associated with ypT0 or survival in a balanced cohort homogenously treated with at least 2 cycles of NAC (7). Conversely, researchers at Columbia University found that patients with MIBC who had a visibly complete TURBT prior to NAC experienced higher 5-year cancer-specific survival (85% versus 50%, P=0.001) and lower rates of ≥ypT2 disease at cystectomy (6). The present study adds to these conflicting findings by contributing to the preponderance of data suggesting the absence of a significant cancer-specific survival benefit associated with complete TURBT prior to NAC and RC. In this patient population, the decision to pursue a repeat TURBT must also be weighed against its potential harms - including delay in initiation of systemic therapy or the potential for adverse oncologic outcomes or potential increase in morbidity of subsequent RC (19–21).

As with all retrospective studies, ours is not without limitations and inherent biases. Retrospective data capture and reliance on operative reports to define the exposure of interest is subject to measurement bias. There was invariably selection bias in the initial decision to offer patients NAC as well as in whom a complete TURBT was performed that could not be complete adjusted for. There are several notable unmeasured potential confounders including tumor multifocality, size, and history of prior non-muscle invasive disease that were not reliably available but could have influenced results. The observed improvement in OS among patients who had a complete TURBT despite similar outcomes in pathologic downstaging, RFS and CSS is likely an example of this residual confounding as unmeasured medical confounders may have influenced the decision to complete a maximal TURBT. As this was a single-center study, findings may not be generalizable to all practice settings.

Ultimately, prospective data will be needed to definitively answer this question. Important insights are likely to come from the ongoing BladderPath study – a clinical trial randomizing patients to complete TURBT versus in-office biopsy prior to NAC and RC which is currently in progress (22). However, until these data are known, we must rely on retrospective series such as the one reported here to inform clinical decision-making.

## CONCLUSIONS

Herein, we observed that a visibly complete TURBT was not associated with pathologic downstaging, recurrence-free or cancer-specific survival following NAC and RC. During each TURBT, a visibly complete resection should continue to be attempted whenever possible in accordance with clinical practice guidelines. However, these data do not support the need for repeat TURBT to achieve a visibly complete resection in situations where NAC and RC are planned.

## APPENDIX

**Supplemental Table 1:** Logistic regression model coefficients for evaluating the association of TURBT completeness with pathologic response at cystectomy.

	Associations with Downstaging at RC		Associations with Complete Response at RC	
Variable	β±SE	OR (95% CI)	β±SE	OR (95% CI)
*Intercept*	-1.72±1.35		-1.48±0.58	
Incomplete TURBT	-0.03±0.43	0.97 (0.42-2.26)	0.21±0.46	1.24 (0.49-3.01)
Male gender			1.04±0.60	2.84 (0.96-10.50)
BMI at initial TURBT (+5 kg/m2)				
ECOG≥ 1 at initial TURBT	-0.65±0.40	0.52 (0.24-1.12)		
Preoperative hydronephrosis at initial TURBT	-1.82±0.44	0.16 (0.06-0.37)	-1.65±0.52	0.19 (0.06-0.49)
Clinical stage at initial TURBT				
Histology (other vs. urothelial) at initial TURBT				
Had second TURBT				
Cycles of chemotherapy (+1 cycle)				
Gem/Cis vs. MVAC/Other				
Age at RC (+10y)	0.31±0.20	1.36 (0.92-2.06)		
Days from initial TURBT to cystectomy (doubling)				

RC=radical cystectomy; β=coefficient; SE=standard error; OR=odds ratio; CI=confidence interval.

The final multivariable models for examining associations of TURBT completeness (incomplete vs. complete) with the separate pathologic response outcomes, downstaging and complete response, are shown. Each of the above models was obtained by first fitting a multivariable logistic regression model with all the above variables as covariates. The variable with the highest p-value (not including incomplete TURBT) was removed from the model in a backward stepwise fashion until there was no longer a reduction in the Akaike Information Criterion.

**Supplemental Table 2:** Cox proportional hazards regression model coefficients for evaluating the association of TURBT completeness with survival outcomes after cystectomy.

		Death, Any Cause		Cancer-Related Death		Recurrence	
Variable		β±SE	HR (95% CI)	β±SE	HR (95% CI)	β±SE	HR (95% CI)
Incomplete TURBT		0.57±0.27	1.76 (1.04-2.98)	0.38±0.38	1.47 (0.70-3.10)	-0.11±0.33	0.90 (0.47-1.71)
Male gender				1.00±0.51	2.73 (1.00-7.41)		
BMI at initial TURBT (+5 kg/m^2^)				-0.26±0.18	0.77 (0.54-1.10)	-0.33±0.14	0.72 (0.54-0.95)
ECOG status at initial TURBT ≥ 1		0.67±0.26	1.96 (1.18-3.25)	0.63±0.38	1.87 (0.90-3.91)		
Preoperative hydronephrosis at initial TURBT				0.87±0.38	2.39 (1.14-5.03)		
Clinical stage at initial TURBT							
Histology (other vs. urothelial) at initial TURBT							
Had second TURBT				-0.86±0.45	0.42 (0.17-1.03)		
Cycles of chemotherapy (+1 cycle)							
Gem/Cis vs. MVAC/Other							
Age at RC (+10y)				-0.29±0.21	0.75 (0.50-1.12)	-0.29±0.14	0.75 (0.57-0.99)
Days from initial TURBT to cystectomy (doubling)				0.75±0.38	2.13 (1.01-4.46)	0.59±0.25	1.80 (1.09-2.97)
Open vs. robotic approach							
**Pathologic T stage (+1 stage)**		**0.42±0.10**		**0.50±0.16**		**0.59±0.11**	
	T0		1.00 (reference)		1.00 (reference)		1.00 (reference)
	Ta/Tis/T1		1.12 (0.44-2.87)		8.56 (1.00-73.3)		2.89 (1.02-8.18)
	T2		1.84 (0.80-4.20)		13.39 (1.69-106)		4.04 (1.55-10.55)
	T3		2.93 (1.34-6.42)		15.27 (1.85-126)		6.84 (2.64-17.72)
	T4		4.95 (2.12-11.52)		18.75 (1.95-180)		12.98 (4.87-34.59)
Pathologic N+							
Positive soft tissue margin				1.25±0.48	3.49 (1.35-9.02)		

RC=radical cystectomy; β=coefficient; SE=standard error; HR=hazard ratio; CI=confidence interval.

The final multivariable models for examining associations of TURBT completeness (incomplete vs. complete) with the survival outcomes (all-cause death, cancer-related death, and recurrence) are shown. Each of the above models was obtained by first fitting a multivariable Cox proportional hazards regression model with all the above variables as covariates. The variable with the highest p-value (not including incomplete TURBT) was removed from the model in a backward stepwise fashion until there was no longer a reduction in the Akaike Information Criterion. The multivariable models included pathologic T stage as a numeric variable (0=T0, 1=Ta/Tis/T1, 2=T2, 3=T3, 4=T4), however the models were refit with pathologic T stage as a categorical variable for purposes of estimating hazard ratios for each stage with T0 as the reference.
